# The Disruption of an OxyR-Like Protein Impairs Intracellular Magnetite Biomineralization in *Magnetospirillum gryphiswaldense* MSR-1

**DOI:** 10.3389/fmicb.2017.00208

**Published:** 2017-02-14

**Authors:** Yunpeng Zhang, Tong Wen, Fangfang Guo, Yuanyuan Geng, Junquan Liu, Tao Peng, Guohua Guan, Jiesheng Tian, Ying Li, Jilun Li, Jing Ju, Wei Jiang

**Affiliations:** ^1^State Key Laboratory of Agro-Biotechnology and Ministry of Agriculture Key Laboratory of Soil Microbiology, College of Biological Sciences, China Agricultural UniversityBeijing, China; ^2^France-China Bio-Mineralization and Nano-Structures LaboratoryBeijing, China; ^3^Beijing Key Laboratory for Prevention and Control of Infectious Diseases in Livestock and Poultry, Institute of Animal Husbandry and Veterinary Medicine, Beijing Academy of Agriculture and Forestry SciencesBeijing, China; ^4^College of Chemistry and Molecular Engineering, Peking UniversityBeijing, China

**Keywords:** OxyR-like protein, magnetosome formation, *Magnetospirillum gryphiswaldense* MSR-1, carbon metabolism, biomineralization

## Abstract

Magnetotactic bacteria synthesize intracellular membrane-enveloped magnetite bodies known as magnetosomes which have been applied in biotechnology and medicine. A series of proteins involved in ferric ion transport and redox required for magnetite formation have been identified but the knowledge of magnetosome biomineralization remains very limited. Here, we identify a novel OxyR homolog (named OxyR-Like), the disruption of which resulted in low ferromagnetism and disfigured nano-sized iron oxide crystals. High resolution-transmission electron microscopy showed that these nanoparticles are mainly composed of magnetite accompanied with ferric oxide including α-Fe_2_O_3_ and 𝜀-Fe_2_O_3_. Electrophoretic mobility shift assay and DNase I footprinting showed that OxyR-Like binds the conserved 5′-GATA-N{9}-TATC-3′ region within the promoter of pyruvate dehydrogenase (*pdh*) complex operon. Quantitative real-time reverse transcriptase PCR indicated that not only the expression of *pdh* operon but also genes related to magnetosomes biosynthesis and tricarboxylic acid cycle decreased dramatically, suggesting a link between carbon metabolism and magnetosome formation. Taken together, our results show that OxyR-Like plays a key role in magnetosomes formation.

## Introduction

Magnetotactic bacteria (MTB) synthesize specialized intracellular membrane-bound organelles called magnetosomes that are arranged in chains by the action of the skeleton-like protein MamK and its binding partner MamJ (Bazylinski and Frankel, 2004; Komeili et al., 2006; Scheffel et al., 2006; Jogler and Schüler, 2009; Komeili, 2012). The iron crystals in most MTB are composed of magnetic magnetite (Fe_3_O_4_), but also of iron sulfide greigite (Fe_3_S_4_) in some bacteria from marine environments (Bazylinski et al., 1995). Magnetite particles have also been reported in higher organisms, including bees, pigeons and human brains (Gould et al., 1978; Walcott et al., 1979; Kirschvink et al., 1992; Komeili, 2007); however, the mechanism of biomineralization of these particles mostly remains unknown.

It has been proven that magnetite biomineralization is mainly regulated by genes that encode proteins involved in ferric ion transport and redox, magnetosome vesicle biosynthesis and alignment, most of which are localized to a large unstable genomic region spanning 80–150 kb in length in different MTBs called the magnetosome island (MAI) (Grunberg et al., 2004; Murat et al., 2010). Some genes in the MAI encode proteins related to magnetosome synthesis, viz., MamA and MamE, which are involved in the sorting and activation of *mam* genes (Komeili et al., 2004; Quinlan et al., 2011; Zeytuni et al., 2011; Hershey et al., 2016; Nguyen et al., 2016; Raschdorf et al., 2016); MamL, Q, B, I, E, M, and O, which function in the invagination of magnetosome vesicles (Raschdorf et al., 2016); and MamY, which influences the shape of magnetosome vesicles (Tanaka et al., 2010). Furthermore, the cation diffusion facilitator family proteins MamB and MamM participate in ferric or ferrous ion transport (Grunberg et al., 2004; Faivre and Schuüler, 2008; Uebe et al., 2011). The interaction of MamJ and MamK maintains the stability of the magnetosome chain (Komeili et al., 2006; Pradel et al., 2006; Scheffel et al., 2006; Scheffel and Schüler, 2007; Draper et al., 2011). MamGFDC and FtsZ-like protein regulate the size of magnetosomes (Scheffel et al., 2008; Ding et al., 2010; Murat et al., 2012). Mms6 plays a key role in the magnetite crystallization process *in vitro* (Arakaki et al., 2003; Tanaka et al., 2011). MamP, a *c*-type cytochrome protein, acts as an iron oxidase that controls the formation of iron(III) ferrihydrite (Siponen et al., 2013; Taoka et al., 2014; Jones et al., 2015). Deletion of *feoB1* reduces the size and quantity of magnetosomes and decreases ferrous ion uptake of the cell (Rong et al., 2008).

In addition to the integrant genes described above, many genes located outside the MAI also contribute significantly to this process. For example, mutation of *fur* or *fur-like* in *Magnetospirillum gryphiswaldense* MSR-1 affects iron homeostasis and increases tolerance to H_2_O_2_ (Uebe et al., 2010; Qi et al., 2012). Moreover, of the six ferric reductases in MSR-1, mutants of *fer5* and *fer6* display defective magnetosomes (Zhang et al., 2013). A series of reductases, oxidases and oxygen sensors are involved in magnetosome biomineralization in MSR-1 (Li et al., 2013, 2014a,b; Jones et al., 2015). The biosynthesis of magnetosomes is also energy-dependent because ATPase is involved in iron ion uptake and transport (Suzuki et al., 2007), and mutation of *crp*, a global regulator that is responsible for energy metabolism, impairs magnetosome synthesis (Wen et al., 2016). Collectively, these studies suggest that the biomineralization process of magnetosomes in MTB is complex and involves numerous regulatory pathways, suggesting that the formation of magnetosomes is regulated by global transcriptional regulators.

To explore the regulatory network of magnetosome biosynthesis in *M. gryphiswaldense* MSR-1, a member of the LysR-type transcriptional regulator (LTTR) family named OxyR-Like (MGMSRv-2-2107) was characterized in MSR-1, and the role of OxyR-Like in the biomineralization of magnetites was studied. The LTTR family contains more than 800 homologous proteins and is widely present among bacteria (Schell, 1993). For most of the LTTR family members studied, after stimulation by an inducer, two dimers binding at different positions within the DNA binding region interact with each other to form a tetrameric protein, thereby altering the transcriptional activity (David and Jan Roelof, 2004). Members of the LTTR family are involved in the regulation of various genes related to metabolism, stress response and cell division in various species (Farr and Kogoma, 1991; Brice et al., 2007; Lu et al., 2007).

The disruption of *oxyR-Like* in MSR-1 resulted in low magnetism and intracellular iron content as well as abnormal magnetosome microstructure. Interestingly, high-resolution transmission electron microscopy (HRTEM) analysis revealed that a large percentage of these irregular magnetosomes were composed of α-Fe_2_O_3_ or *𝜀*-Fe_2_O_3_, an unstable phase under normal conditions. The regulatory mechanism of OxyR-Like was therefore also explored to explain its role in magnetosome formation. *In vitro* binding assays between OxyR-Like and DNA probes indicated that the expression of the pyruvate dehydrogenase (*pdh*) operon was regulated by OxyR-Like. Quantitative real-time reverse transcriptase PCR (qRT-PCR) analysis further indicated that in the mutated *oxyR-Like* strain, the expression of genes located on the *pdh* operon, as well as the genes related to the tricarboxylic acid (TCA) cycle and located on the MAI, decreased dramatically.

In summary, these results clearly demonstrate that the LTTR family regulator OxyR-Like, which controls the transcription of the *pdh* operon, plays a key role in the magnetosome formation.

## Materials and Methods

### Bacteria and Growth Conditions

The bacterial strains and plasmids used in this study are described in Supplementary Table S1. *M. gryphiswaldense* MSR-1 was cultured in sodium lactate medium (SLM) at 30°C as described previously (Guo et al., 2012). The medium contained (per liter of deionized water) 1.5 g sodium lactate, 0.4 g NH_4_Cl, 0.1 g yeast extract, 0.5 g K_2_HPO_4_, 0.1 g MgSO_4_⋅ 7H_2_O, 0.05 g sodium thioglycolate and 5 mL of trace element mixture. The iron source, ferric citrate, was added at a final concentration of 60 μM after autoclaving. For conjugation, *M. gryphiswaldense* strains were cultured in selection medium, in which NH_4_Cl and yeast extract were substituted with sodium glutamate (4 g per liter) (Guo et al., 2012). The strains were cultured in 250 mL serum bottles containing 100 mL of medium with shaking at 100 rpm. As the cell density increased, microaerobic conditions developed in the medium because of oxygen consumption. *Escherichia coli* strains were cultured in Luria broth (LB) at 37°C. Antibiotics were used at the following concentrations: for *E. coli*: 100 μg/mL ampicillin (Amp), 25 μg/mL chloramphenicol (Cm) and 20 μg/mL gentamicin (Gm); for MSR-1: 5 μg/mL nalidixic acid (Nx), 5 μg/mL Cm and 5 μg/mL Gm.

### Construction of the *oxyR-Like^-^* Mutant Strain

The primers used in this study are listed in Supplementary Table S2. Fragments containing the upstream region (961 bp) and downstream region (1064 bp) of *oxyR-Like* were amplified using the primer pairs oxyR-Like-uf/oxyR-Like-ur and oxyR-Like-df/oxyR-Like-dr, respectively. The Gm cassette was digested from the pUC-Gm vector with *Sac*I. The three fragments were fused by cloning them into the *Hin*dIII and *Bam*HI sites of the pSUP202 vector to yield pSU-OxyR-Like. The pSU-OxyR-Like plasmid was introduced into wild-type MSR-1 by biparental conjugation, followed by screening for Gm^r^ Cm^s^ colonies as previously described (Rong et al., 2008). Double crossover was confirmed by PCR. The resulting mutant strain was termed *oxyR-Like^-^*.

### Construction of the *oxyR-Like^-^* Complementary Strain

The primers coxyR-Like-f and coxyR-Like-r, which contained restriction sites for *Hin*dIII and *Bam*HI, were used for cloning of the *oxyR-Like* gene. The primers kanp-f and kanp-r, which contained restriction sites for *Sac*I and *Bgl*II (1141 bp), were used for cloning of the kanamycin (kan) promoter (from plasmid pUC4K). The amplified fragments were ligated to the pMD18-T simple vector (code D104A; TaKaRa Biotechnology, Dalian, China) and digested with *Bam*HI/*Hin*dIII or *Sac*I/*Bgl*II, repectively. The resulting *Bam*HI-*Hin*dIII oxyR and *Sac*I-*Bgl*II kan promoter fragments were ligated into the *Sac*I-*Hin*dIII sites of pPR9TT to generate pPROxyR-Like. pPROxyR-Like was introduced into the *oxyR-Like^-^* mutant by biparental conjugation, and transconjugants were screened for Gm^r^ Cm^r^ colonies. The presence of the intact *oxyR-Like* gene was confirmed by PCR. The complementary strain of the *oxyR-Like^-^* mutant was termed *oxyR-Like^-^* + *oxyR-Like*. To guarantee the parallelism of the three strains, pPR9TT with no *oxyR-Like* fragment was also introduced into wild type MSR-1 (termed Wild type) and the *oxyR-Like^-^* mutant.

### Expression and Purification of 6His-OxyR-Like

The *oxyR-Like* gene was cloned by PCR from MSR-1 using the primers OxyR-Like-P-f and OxyR-Like-P-r, which contained *Eco*RI and *Xho*I sites, respectively. The amplified fragment was ligated to pMD18-T for sequencing, digested with *Eco*RI and *Xho*I and ligated to digested pET28a (+) to construct the plasmid pET-28a-oxyR-Like. pET-28a-oxyR-Like was transformed into the *E. coli* strain BL-21, which contains a *lacUV* promoter-driven T7 RNA polymerase. Isopropyl-β- D-thiogalactoside (IPTG) at a final concentration of 300 μM was used to induce the expression of 6His-OxyR-Like in LB medium. The cells were then sonicated and centrifuged. The supernatants containing 6His-OxyR-Like were applied to a nickel-nitrilotriacetic acid-agarose (Ni-NTA) column (code 70666-3; Novagen, Germany) and equilibrated with buffer (50 mM Tris-HCl (pH 8.0), 300 mM NaCl, 1 mM imidazole). Proteins conjugated with Ni-NTA were eluted with 200 mM imidazole buffer containing 50 mM NaH_2_PO_4_ and 300 mM NaCl. The purified fusion protein was detected by sodium dodecyl sulfate polyacrylamide gel electrophoresis (SDS-PAGE).

### Western Blotting

For SDS-PAGE, 16 μL of cell extract from the sonicated samples (3 s/6 s, 200 W, 150 repetitions) was mixed with 4 μL of 5× sample loading buffer and incubated at 100°C for 10 min. The mixture was separated on a 5% stacking gel and 15% resolving gel, then transferred onto polyvinylidene fluoride (PVDF) membranes and analyzed by western blotting using polyclonal antiserum raised against 6His-OxyR-Like. The secondary antibody was developed with goat anti-mouse IgG antibody conjugated with alkaline phosphatase (Sigma–Aldrich, Saint Louis, MO, USA). The membrane was visualized using a BCIP/NBT (5-bromo-4-chloro-3-indolyl-phosphate/nitro blue tetrazolium) chromogenic reagent kit (Tiangen, China) according to the manufacturer’s instructions.

### Cell Growth and Magnetic Response Curves

All strains were grown synchronously in SLM at 30°C for 30 h. The optical density (OD_600_) was measured using a UV-visible spectrophotometer (UNICO2100; UNICO Instrument Co., Shanghai, China). The coefficient of magnetism (Cmag) values were calculated from measurements of the maximal and minimal scattering intensities (Zhao et al., 2007). The OD_600_ and Cmag values were measured every 3 h, and OD_600_ and Cmag curves were constructed.

### Transmission Electron Microscope (TEM) Measurements and Cryo-ultramicrotomy

The bacterial strains used for the TEM measurements were grown in SLM (supplemented with 60 μM ferric citrate) at 30°C for 24 h until cell reached the stationary phase. The cells were rinsed twice with double-distilled H_2_O, and the suspensions were adsorbed onto copper grids and observed by TEM. The structural details of the nanoparticles in the three types of cells were determined by the HRTEM method using a JEM-2100F (JEOL Ltd., Tokyo, Japan). The machine was operated at 200 kV and was equipped with a field emission gun, ultra-high-resolution pole piece and ultrathin window JEOL detector. HRTEM images were obtained with an OSIS CANTEGA CCD camera. The crystals’ structural parameters were obtained by fast Fourier transform (FFT) analyses. Ultrathin cryosections were obtained by fixing a small pellet of each strain in 2% glutaraldehyde in phosphate-buffered saline (PBS) overnight at 4°C. After centrifugation, the samples were embedded in 10% gelatin for 1 h at 37°C and then overnight at 4°C. The blocks were dispersed in 2.3 M sucrose in PBS for 1 h at 37°C and then overnight at 4°C. Ultrathin cryosections were obtained from several blocks and stained with methyl cellulose and uranylacetate for 5 min.

### Residual Iron in Medium and Intracellular Iron Content Analysis

Each strain was cultured microaerobically at 30°C in SLM (containing 60 μM ferric citrate). After reaching stationary phase, the cells were harvested by centrifugation. The total iron ions in the supernatant were measured by ferrozine assay for residual iron content analysis (Stookey, 1970). The cell pellets were washed three times with 20 mM Tris-HCl buffer containing 4 mM EDTA (pH 7.4). The pellets were dried to constant weight at 60°C, resuspended in 1 mL of nitric acid and incubated at 100°C for 3 h. The iron content was assayed using an atomic absorption spectrometer (Optima 5300DV, PerkinElmer, Waltham, MA).

### Electrophoretic Mobility Shift Assay (EMSA) and DNase I Footprinting

**FIGURE 1:**
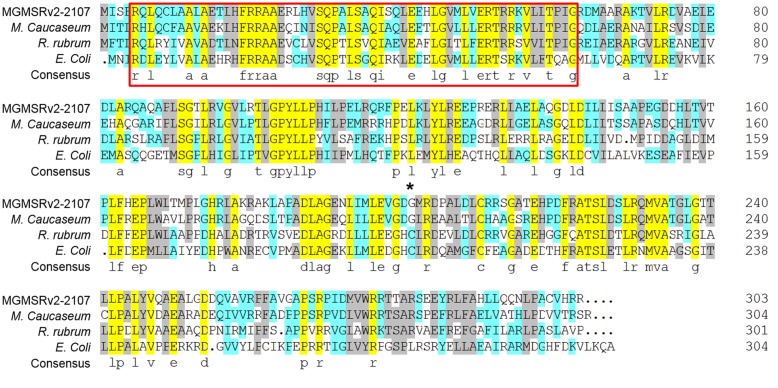
**Multiple alignments of the OxyR-Like protein, LysR family transcriptional regulator from *Magnetospirillum caucaseum, Rhodospirillum rubrum*, and LysR family DNA-binding transcriptional regulator OxyR from *E. coli.*** Conserved amino acid residues among these four proteins are highlighted by light purple, the proposed helix-turn-helix DNA-binding domain is marked by red, and the non-conserved reactive Cys residues between OxyR from *Escherichia coli* and OxyR-Like from MSR-1 are marked by an asterisk.

Electrophoretic mobility shift assay (EMSAs) were performed using a DIG Gel Shift Kit, 2nd Generation (Roche). The DNA probes were amplified by PCR (the primers are listed in Supplementary Table S2) and labeled with digoxigenin (DIG) at the 3′ ends following the manufacturer’s instructions. The probes were then mixed with the appropriate amount of 6His-OxyR-Like protein (stated in **Figure 5**) and 1 μL poly [d(I-C)] distributed in binding buffer to a final volume of 20 μL; the mixture was incubated at 25°C for 30 min and mixed with 5 μL 5× loading buffer with bromophenol blue. Protein-bound and free DNA were separated by electrophoresis on non-denaturing 5% polyacrylamide gels in 0.5× TBE (0.5× TBE buffer contains 5.4 g Tris base, 2.75 g boric acid and 2 ml 0.5 M pH 8.0 EDTA per liter of deionized water) running buffer and transferred from the gels onto a nylon membrane by electroblotting. After baking for 10 min at 80°C, the membrane was exposed to UV radiation at 256-nm for 10 min to cross-link the DNA fragments and the membrane. Chemiluminescence detection was performed according to the manufacturer’s instructions, and the membranes were exposed to X-ray film (Fuji) for 15–30 min.

To identify binding sites of the OxyR-Like protein in the intergenic region other than its own promotor and the *pdh* operon promotor, a non-radiochemical capillary electrophoresis method was used for DNase I footprinting. Fluorescence-labeled DNA fragments were amplified by PCR. The resulting DNA fragments covered the entire intergenic region. After purification using a Gel DNA Purification Kit (Tiangen, Beijing, China), the labeled DNA fragments (1000 ng) and appropriate concentrations of 6His-OxyR-Like protein were added to a final reaction volume of 25 μL and incubated for 30 min at 25°C. DNase I digestion (0.1 units) was performed for an appropriate duration at 37°C and stopped by the addition of EDTA at a final concentration of 50 mM. The reaction mixture was heated to 80°C for 10 min to completely inactivate DNase I. The samples were subjected to phenol-chloroform extraction, ethanol precipitation and capillary electrophoresis by loading into an Applied Biosystems 3730 DNA Genetic Analyzer together with the internal-lane size standard ROX-500 (Applied Biosystems). The electropherograms were analyzed using the GeneMarker program, v1.8 (Applied Biosystems).

### Quantitative Real-Time Reverse Transcriptase PCR (qRT-PCR)

Total RNA of a samples taken at 24 h after inoculation was extracted using TRIzol reagent (Tiangen, Beijing, China) following the manufacturer’s instructions. The remaining DNA in the RNA was digested using DNase I (Takara, Shiga, Japan). cDNA was synthesized by reverse transcription using M-MLV reverse transcriptase (Promega Corp., San Luis Obispo, CA, USA), dNTPs and random primers (Takara, Shiga, Japan) according to the manufacturers’ instructions. qRT-PCR analysis was then performed to determine the transcription levels of the selected *pdh* operon, the tricarboxylic acid cycle-related genes and the *mam* genes using the obtained cDNA as a template and the corresponding primers. The *rpoA* gene was used as a positive internal reference gene in this study; the *rpoA* gene encodes the DNA-directed RNA polymerase alpha chain in MSR-1(Ritz et al., 2009; Wen et al., 2016). The qRT-PCR assay was performed using a LightCycler 480 Instrument II (Roche, South San Francisco, CA, USA) and the SYBR Green I Master kit (Roche), according to the manufacturer’s recommendations. The total volume of each reaction was 20 μL, the template cDNA content in each reaction mixture was approximately 50 ng, and the concentration of each oligonucleotide was 0.5 μM. The cycling parameters for qRT-PCR were as follows: initial denaturation at 95°C for 10 min, followed by 45 cycles of denaturation at 95°C for 15 s, annealing at 62°C for 5 s and extension at 72°C for 15 s. Finally, the transcription level of each gene tested was determined according to the threshold cycle (ΔC_T_) method, which is an improvement of the Livak method where ΔC_T_ = C_T_ (reference gene) –C_T_ (target gene).

## Results

### Construction and Characterization of the *oxyR-Like* Mutant of MSR-1

The *oxyR*-*Like* gene of MSR-1 (locus tag MGMSRv-2-2107 or MGR-2168) is 909 bp and encodes a protein of 303 residues. Although the sequence of MGMSRv-2-2107 exhibits high homology with that of the OxyR protein from *E. coli*, a functional position, Cys201, was absent (**Figure 1**, asterisk). The absence of Cys201 hints that this protein has a new function in MSR-1. Multiple sequence alignments between OxyR-Like (MGMSRv-2-2107) and three other homologous proteins from *Magnetospirillum caucaseum* (LysR family transcriptional regulator, NCBI reference sequence: WP_008619764.1), *Rhodospirillum rubrum* (LysR family transcriptional regulator, NCBI reference sequence: WP_011390592.1) and *E. coli* (LysR family DNA-binding transcriptional regulator OxyR, NCBI reference sequence: WP_033556234.1) revealed that two functional domains are highly conserved among these four proteins, namely the N-terminal LysR family bacterial regulatory helix-turn-helix domain, belonging to a large family that primarily function as sequence-specific DNA-binding domains (**Figure 1**, red frame), and the LysR substrate binding domain from amino acid residues 87 to 297 (**Figure 1**). The sequence alignment results also suggested that the protein region composed of these two conserved domains has high homology with the aminoethylphosphonate catabolism-associated LysR family transcriptional regulator.

To explore the function of *oxyR-Like* in MSR-1, an *oxyR-Like* mutant was first constructed. To generate the *oxyR- Like* disruption mutant, using biparental conjugation, the *oxyR-Like* genomic region of wild-type MSR-1 (Wild type) was replaced with a gentamycin (Gm) resistance sequence. The resulting *oxyR-Like* mutants, termed *oxyR-Like^-^*, were screened by Gm resistance (Supplementary Figure S1A) and further confirmed by PCR (Supplementary Figure S1B). To determine whether any mutant phenotype resulted from *oxyR-Like* deficiency, a complementation strain, termed *oxyR-Like^-^*+ *oxyR-Like*, was constructed and verified by PCR (Supplementary Figure S1B). To measure the expression of OxyR-Like in different strains, His-tagged OxyR-Like was expressed in *E. coli* and purified (Supplementary Figure S2). Polyclonal antibodies were then prepared for western blot analysis. OxyR-Like was subsequently detected in the Wild type and complementation strains but not in *oxyR-Like^-^* (Supplementary Figure S1C), validating these strains for use in subsequent experiments.

### *oxyR-Like^-^* Displays Defective Ferromagnetism and Low Intracellular Iron Content

**FIGURE 2:**
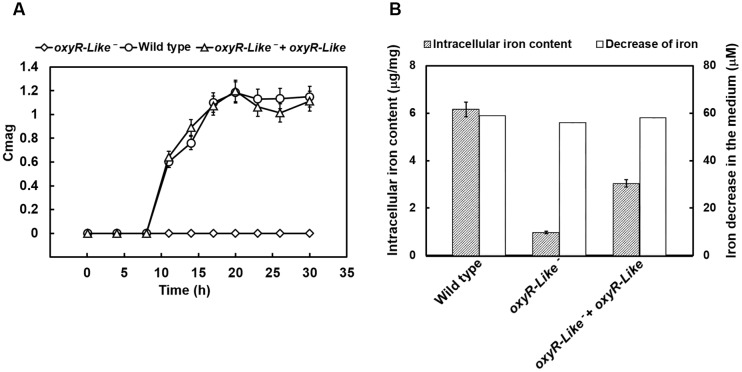
**Magnetism, intracellular iron content, and medium iron content analyses of various strains.** **(A)** Time-resolved magnetism of Wild type, *oxyR-Like^-^* and *oxyR-Like^-^* + *oxyR-Like*. Magnetism detected for each strain was calculated by the coefficient of magnetism (Cmag) as described in the Section “Materials and Methods”. **(B)** Measurements of intracellular iron content in Wild type, *oxyR-Like^-^* and *oxyR-Like^-^* + *oxyR-Like* cells and the corresponding decrease in iron content in the medium. All experiments were independently repeated three times to ensure their reproducibility.

Time-course experiments were conducted to measure the cell growth and magnetic response of all strains. As shown in Supplementary Figure S3, the growth of *oxyR-Like^-^* was slower than that of the Wild type and *oxyR-Like^-^*+ *oxyR-Like* strains. The coefficient of magnetism (Cmag, defined in the Materials and Methods) values indicated that ferromagnetism decreased dramatically in the *oxyR-Like*^-^ deficient mutant, whereas the complementation strain phenocopied the Wild type (**Figure 2A**). The *oxyR-Like^-^* cell pellets were brown, in contrast to the black–gray pellets of the Wild type and the complementation strain, as shown in Supplementary Figure S4. To determine the residual iron content in the medium and the intracellular iron content, all strains were inoculated in SLM containing 60 μM ferric citrate. The corresponding decreases in iron in the medium after 24 h were 58.7, 55.8, and 57.8 μM, respectively, for the three strains. These changes were not significantly different, as shown in **Figure 2B**. However, the intracellular iron content in Wild type cells (6.15 ± 0.17 μg/mg) and *oxyR-Like^-^* + *oxyR-Like* cells (3.04 ± 0.05 μg/mg) was ∼6.28- and 3.10-fold higher, respectively, than that in *oxyR-Like^-^* cells (0.98 ± 0.30 μg/mg) (**Figure 2B**), which was very similar to that of the *crp^-^* mutant strain, another non-magnetic mutant (Wen et al., 2016).

### Magnetosome Particles Are Disfigured in the *oxyR-Like^-^* Strain

**FIGURE 3:**
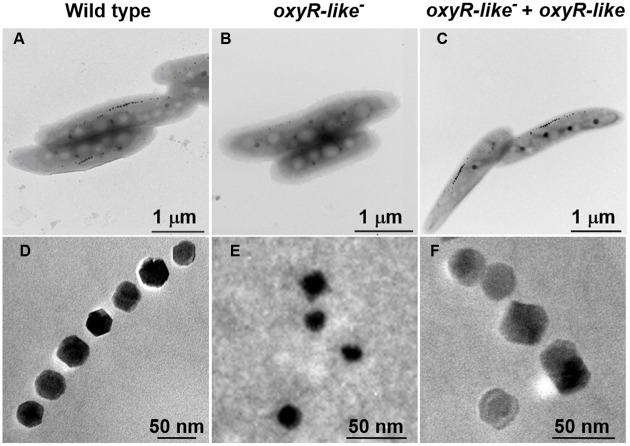
**Magnetosome morphology observations of various strains.** Magnetosome arrangement and morphology of Wild type **(A,D)**, *oxyR-Like^-^* **(B,E)** and *oxyR-Like^-^* + *oxyR-Like* **(C,F)** as assessed by transmission electron microscopy (TEM).

To determine why *oxyR-Like^-^* had low ferromagnetic signals and how the intracellular iron reduction occurred, cells of all strains were observed using TEM. Approximately 30 randomly selected cells from each strain were examined to analyze the morphology and diameter of the magnetosomes. The iron oxide nanoparticles in Wild type cells had a regular cubo-octahedral shape with a diameter of approximately 30-40 nm and were arranged in a line (**Figure 3**). However, the nonmagnetic *oxyR-Like^-^* mutant synthesized disfigured magnetosomes that were no longer arranged in a line (**Figure 3**). Magnetosome membranes were not obviously affected in the mutant (Supplementary Figure S5). The magnetosomes in the complementation strain had a nearly identical particle size and arrangement as those in the Wild type (**Figure 3**), indicating that the complementation strain recovered the ability to synthesize magnetosomes. The diameter of some *oxyR-Like^-^* crystals was ∼50% of that of the Wild type crystals (**Figure 3** and Supplementary Figure S6), and their morphology was defective. The total number of magnetosomes in *oxyR-Like^-^* cells was approximately 3-fold higher than that in *oxyR-Like^-^* + *oxyR-Like* and Wild type cells.

**FIGURE 4:**
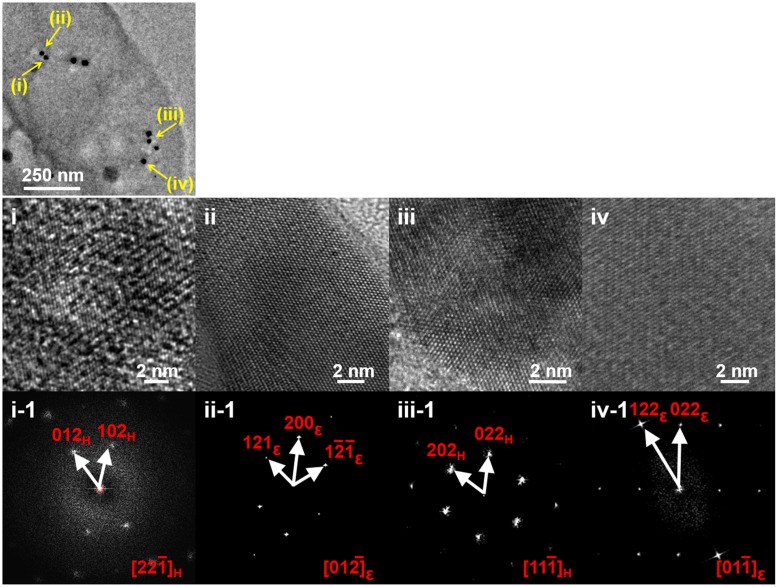
**High-resolution transmission electron microscopy (HRTEM) and fast Fourier transform (FFT) analyses of intracellular magnetosomes from *oxyR-Like^-^*.** Distribution of the scattered particles from *oxyR-Like^-^* as determined by HRTEM and FFT; (i–iv) HRTEM images and corresponding FFTs of particles (i)-(iv) marked by yellow arrows show various Fe(III) oxide phases: *𝜀*-Fe_2_O_3_ (ii, iv) and *𝜀*-Fe_2_O_3_ (i, iii).

After the primary characterization of magnetosomes formed by *oxyR-Like^-^*, HRTEM imaging techniques were used to determine the structure of these disfigured nanoparticles and spatially resolve the mechanism of biomineralization. Wild type cells exhibited a single crystal, described as a pure magnetite structure (Supplementary Figure S7A; Supplementary Table S3). The corresponding fast FFT pattern was uniquely indexed using cubic-phase magnetite with the zone axis [11 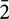]*_M_*. Our notation convention using *M*, *𝜀*, and *H* subscripts denotes the plane and orientation indices in Fe_3_O_4_ (magnetite), α-Fe_2_O_3_ and 𝜀-Fe_2_O_3_ (hematite) phases, respectively. Similarly, the nanoparticles in the *oxyR-Like^-^* + *oxyR-Like* cells were indexed using the magnetite structure with the zone axis [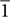 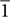 6]_M_ (Supplementary Figure S7B, Supplementary Table S3). By contrast, the magnetosomes in the *oxyR-Like^-^* strain exhibited diverse crystal structures. In addition to the larger and more regular particles, which corresponded to magnetite, the particles indicated by the yellow arrows in **Figure 4** were characterized as α-Fe_2_O_3_ (hematite) and 𝜀-Fe_2_O_3_ (**Figure 4**i–iv, Supplementary Table S3). In **Figure 4**i, the HRTEM image and FFT analysis of particle (i) correspond to the crystal fringes and electron diffractions of hematite with zone axis [22 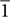]*_H_*. Particle (ii) in **Figure 4**ii was *𝜀*-Fe_2_O_3_ with zone axis [01 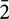]*𝜀*. Similarly, particles (iii) and (iv) in **Figure 4** were indexed as hematite and *𝜀*-Fe_2_O_3_ (**Figure 4**iii, iv), respectively. These results indicated that the disruption of *oxyR-Like* impaired the magnetosome formation process.

### OxyR-Like Regulates the Expression of the *pdh* Operon

**FIGURE 5:**
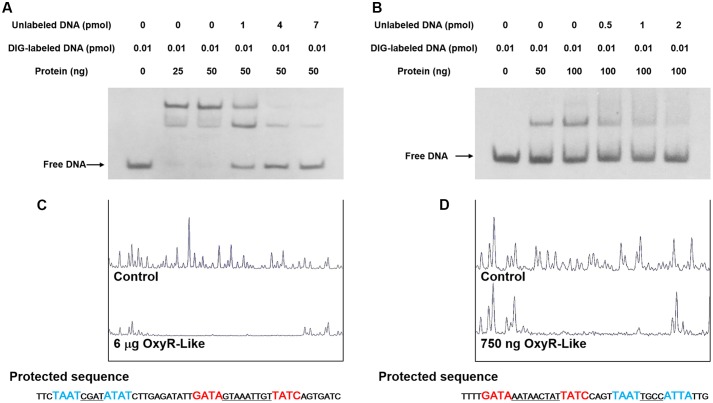
**OxyR-Like specifically binds to the *oxyR-Like* and *pdh* operators.** **(A,B)** OxyR-Like specifically binds to the *oxyR-Like* **(A)** and *pdh* **(B)** promoters, and the interactions between the protein and DNA were dissociated by unlabeled probe. **(C,D)** The *oxyR-Like* **(C)** and *pdh* **(D)** promotor regions were protected by OxyR-Like in the DNase I footprinting assay. All experiments were independently repeated three times to ensure their reproducibility. The black arrows in the figure indicate the positions of free DNA probes.

To investigate the OxyR-Like regulatory mechanism, 6His-OxyR-Like was purified and subjected to EMSAs. In the presence of multiple repeats, 6His-OxyR-Like interacted with its own gene promoter region as well as that of the *pdh* operon (**Figures 5A,B**). The protein-DNA complex was dissociated by unlabeled probe, confirming the specificity of the interaction (**Figures 5A,B**). To further determine the binding site of OxyR-Like with these promoters, DNase I footprinting was performed. OxyR-Like protected two DNA sequences of high similarity from DNase I digestion (**Figures 5C,D**). Both sequences contained the conserved region 5′-GATA-N{9}-TATC-3′ (protected sequence colored red in **Figures 5C,D**), denoted the OxyR-Like box. Another homologous binding site with a high percentage of A/T (protected sequence colored blue in **Figures 5C,D**) corresponded to the second band in the EMSA results.

### Numerous Genes Related to the *pdh* Operon and TCA Cycle Are Down-Regulated by the Disruption of *oxyR-Like*

**FIGURE 6:**
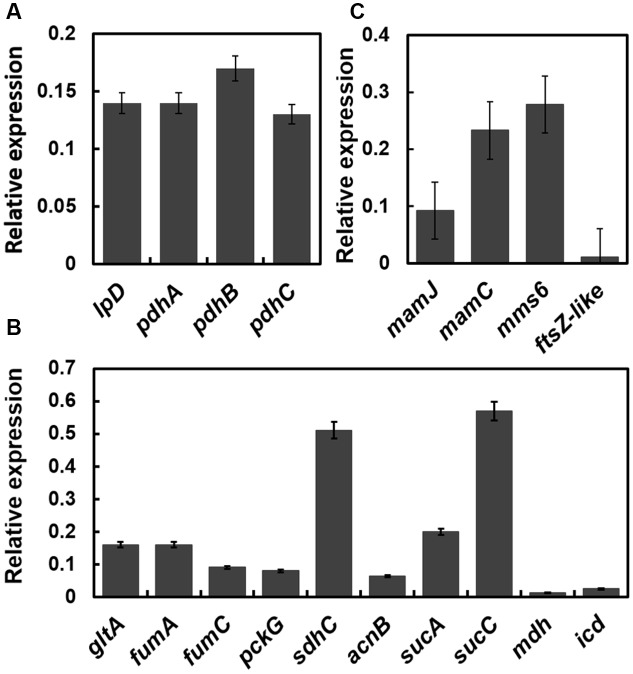
**The transcription levels of genes (*oxyR-Like^-^* mutant versus Wild type) related to pyruvate metabolism, the TCA cycle and the magnetosome island are down-regulated in *oxyR-Like^-^*.** The results were obtained by qRT-PCR, and the relative expression levels of each gene in Wild type were set as 1 and not shown in the data. The reference gene used in this study is *rpoA*, which encodes the RNA polymerase alpha subunit in MSR-1 (locus tag MGMSRv-2-0062). **(A)** Relative expression level of genes located on the *pdh* operator. Genes tested (from left): *pdhA* (pyruvate dehydrogenase E1 component subunit, locus tag MGMSRv-2-0966), *pdhB* (pyruvate dehydrogenase E1 component subunit, locus tag MGMSRv-2-0967), *pdhC* (dihydrolipoyllysine-residue acetyltransferase, locus tag MGMSRv-2-0968), *lpD* (dihydrolipoyl dehydrogenase, locus tag MGMSRv-2-0971). **(B)** Relative expression level of genes related to the TCA cycle. Genes tested (from left): *gltA* (citrate synthase, locus tag MGMSRv-2-1343), *fumA* (fumarase A, locus tag MGMSRv-2-1453), *fumC* (fumarate hydratase, locus tag MGMSRv-2-1684), *pckG* (phosphoenolpyruvate carboxylase, locus tag MGMSRv-2-1769), *sdhC* (succinate dehydrogenase cytochrome b556 subunit, locus tag MGMSRv-2-2218), *acnB* (aconitate hydratase 2, locus tag MGMSRv-2-3179), *sucA* (2-oxoglutarate decarboxylase, locus tag MGMSRv-2-3603), *sucC* (succinyl-CoA synthetase, MGMSRv-2-3605), *mdh* (malate dehydrogenase, locus tag MGMSRv-2-3606), and *icd* (isocitrate dehydrogenase, locus tag MGMSRv-2-4098). **(C)** Relative expression level of genes located on the magnetosome island. All experiments were independently repeated three times to ensure their reproducibility.

Because the *pdh* operon is regulated by OxyR-Like, the expression levels of genes belonging to the *pdh* operon and related to the TCA cycle were determined. The transcription levels of genes within known gene clusters involved in pyruvate metabolism and the TCA cycle were detected by qRT-PCR (**Figures 6A,B**). Most of these genes were down-regulated dramatically, suggesting that the TCA pathway is impaired by the disruption of *oxyR-Like*.

### Magnetosome Island (MAI) Genes are Down-Regulated in the *oxyR-Like^-^* Mutant

To explain the role of OxyR-Like in magnetosome formation in MSR-1, we next focused on the target genes related to magnetosome biosynthesis. The transcriptional levels of genes located within the known gene clusters involved in magnetosome biosynthesis, such as *mamJ* (*mamAB* cluster), *mamC* (*mamGFDC* cluster), *mms6* and *ftsZ-like* (*mamXY* cluster), were detected by qRT-PCR. The results indicated that the transcriptional levels of *mamJ*, *mamC*, *mms6* and *ftsZ-like* in the *oxyR-Like^-^* mutant were reduced by approximately 10.87, 4.29, 3.58, and 90.91-fold, respectively, compared with those in Wild type cells (**Figure 6C**), suggesting that the disruption of *oxyR-Like* impaired the expression of genes located on the MAI.

## Discussion

In MTB, the biomineralization process of magnetosomes is highly complex and is not fully understood. It is regulated and promoted not only by genes located on the MAI, but also by some genes that participate in the cell’s basal metabolism. In this study, it was determined that the disruption of a novel LysR-type transcriptional regulator, OxyR-Like, leads to disfigured magnetosome crystals, suggesting that OxyR-Like plays a key role during the formation of magnetosomes.

The chemical route for the biomineralization of magnetosome magnetite has been studied for decades. In the previous studies, ferrihydrite was identified as a precursor for mature magnetosome formation using Mõssbauer spectroscopy (Faivre et al., 2007), which was further confirmed using Fe K-edge X-ray absorption near edge structure (XANES) and HRTEM analysis in *M. gryphiswaldense* MSR-1 and *Magnetospirillum magneticum* AMB-1(Baumgartner et al., 2013; Fdez-Gubieda et al., 2013). Also, in *Desulfovibrio magneticus* RS-1, FeS was confirmed to be the intermediate for the formation of Fe_3_S_4_, in which process Fe(II) was oxidized to Fe(III) (Baumgartner et al., 2016). Moreover, the findings of *𝜀*-Fe_2_O_3_ in *mamX* and *mamZ* mutant strains raise the possibility that magnetosome magnetite is biomineralized from ferric oxide intermediates (Raschdorf et al., 2013). In this study, we observed the co-existence of *𝜀*-Fe_2_O_3_, *𝜀*-Fe_2_O_3_, and Fe_3_O_4_ in the mutant strain disrupted in *oxyR-Like*. *𝜀*-Fe_2_O_3_ is a thermally unstable phase of ferric oxide that has been reported in plants (McClean et al., 2001). It was also confirmed by previous work that in MTB, magnetite crystals are biomineralized by the transformation of *𝜀*-Fe_2_O_3_ (Baumgartner et al., 2013; Fdez-Gubieda et al., 2013), so it is possible that the intermediates detected during magnetosome maturation consist of various types of ferric oxide more than *𝜀*-Fe_2_O_3_, due to the complex environment of the cell.

In *E. coli* and many other Gram-negative bacteria, OxyR participates in the regulation of the intracellular redox state by controlling the expression of various antioxidant-related genes, such as *katE*, *katG* and *ahpC*. Intracellular reactive oxygen species (ROS) are required for OxyR activation (Pomposiello and Demple, 2001). OxyR is typically activated by the formation of a disulfide bond between Cys-199 and Cys-208, which is induced by the intracellular ROS and leads to the three-dimensional allosteric structure (Jolanta and Kierzek, 2003). However, due to the absence of a Cys residue near Cys-210 in OxyR-Like, the ability to respond to ROS and bind the promoters of these ROS-eliminating genes is lost (Supplementary Figure S8), suggesting that OxyR-Like is a novel LTTR member recognizing a different inducer.

In MTB, the *mam* genes play significant roles during the formation of normal magnetosome magnetite. Such as, the disruption of MamM and MamB resulted in the loss of magnetism as well as the magnetosome vesicle (Murat et al., 2010; Uebe et al., 2011); in *mamC,F, G, H* mutant strains magnetite crystals are getting smaller (Scheffel et al., 2008; Raschdorf et al., 2013; Lohße et al., 2014); and the loss of *mamD* and *mms6* lead to elongated shape of nano crystal (Scheffel et al., 2008; Tanaka et al., 2011). In this study, the expression levels of four representative genes located on the four main MAI gene clusters were greatly decreased by the disruption of *oxyR-Like*, which could be one of the main reasons for the formation of disfigured magnetite crystals. However, the interactions between OxyR-Like and the promoter regions of these clusters were undetected (data not shown), indicating indirect relationship between the protein and the *mam* genes.

On the other hand, the present study also determined by EMSA that OxyR-Like can bind at two positions within the *pdh* operon promoter region, which is consistent with previous work on the LTTR family (David and Jan Roelof, 2004). This result showed that OxyR-Like in MSR-1 may regulate energy metabolism-related genes rather than ROS-eliminating genes (**Figure 5** and Supplementary Figure S8), which further confirmed our previous speculation that OxyR-Like performed a novel function in MSR-1. qRT-PCR assays further demonstrated that the disruption of OxyR-Like leads to dramatic down-regulation of TCA-related genes, potentially leading to a decrease in intracellular ATP and reducing power.

ATP and reducing power are crucial for cell growth. Previous work has indicated that the biomineralization of magnetosomes is energy-dependent in magnetatic bacteria, as a lack of ATP can lead to a decrease in iron content in mutant strain (Suzuki et al., 2007). We speculate that the disruption of *oxyR-Like* removed the normal regulation of the pyruvate dehydrogenase complex, which functions to generate acetyl coenzymeA. Similarly, the low expression level of the genes participating in the TCA cycle further impaired the generation of ATP and reducing power, which maintain the intracellular energy content and redox state, resulting in poor growth. There still exists the interesting observation that the expression of genes located on MAI is greatly impaired, which should be the direct cause of the formation of the disfigured magnetite crystals. And this association between carbon metabolism and MAI gene expression will be explored next.

## Author Contributions

YZ, TW, and FG conducted most of the experiments. WJ, YL, and JL-L conceived the project and designed the experiments. JJ performed HRTEM observations and analyzed the data. TP expressed and purified the 6His-OxyR protein. YG participated in the TEM data analysis. GG, JQ-L, and JT were involved in writing and revising the manuscript.

## Conflict of Interest Statement

The authors declare that the research was conducted in the absence of any commercial or financial relationships that could be construed as a potential conflict of interest.
