# Supplementary material for: The Disruption of an OxyR-Like Protein Impairs Intracellular Magnetite Biomineralization in Magnetospirillum gryphiswaldense MSR-1
Source: Front Microbiol. 2017 Feb 14;8:208. doi: 10.3389/fmicb.2017.00208 (PMC5308003; doi:10.3389/fmicb.2017.00208)
Supplement: Supplementary file 1 [file Presentation_1.pdf]

The disruption of an OxyR-Like protein impairs intracellular magnetite biomineralization in *Magnetospirillum gryphiswaldense* MSR-1

Yunpeng Zhang[1, 4†], Tong Wen[1, 4†], Fangfang Guo[1,3†], Yuanyuan Geng[1, 4], Junquan Liu[1, 4], Tao Peng[1], Guohua Guan[1, 4], Jiesheng Tian[1, 4], Ying Li[1, 4], Jilun Li[1, 4], Jing Ju[2*], Wei Jiang[1, 4*]

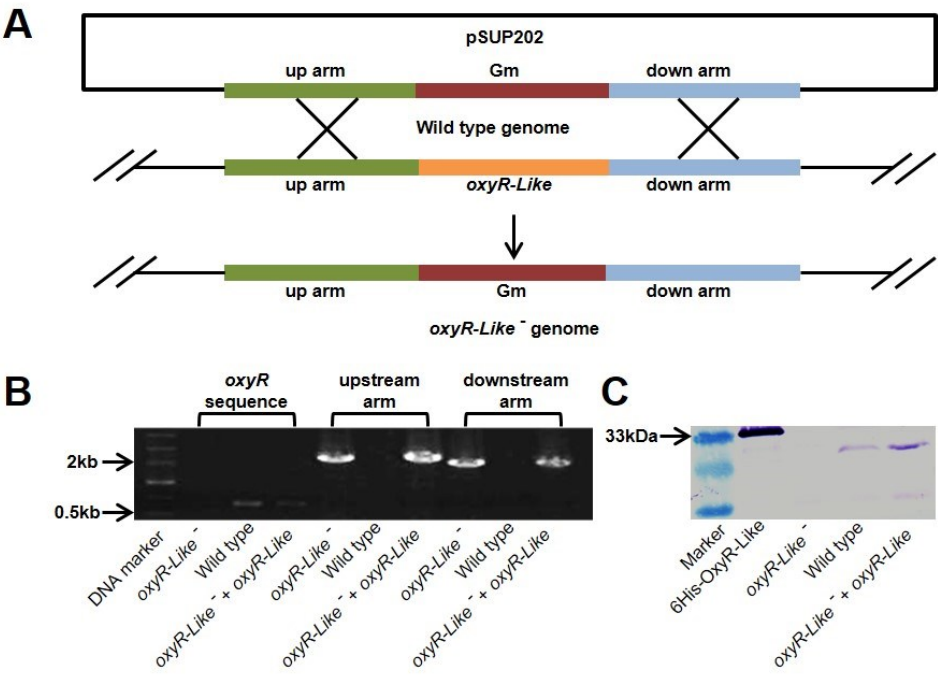

Supplementary Figure 1. Construction and verification of the *oxyR-Like*⁻ mutant and complementary strains. (a) Schematic representation of the *oxyR-Like* disruption. (b) Verification of the *oxyR-Like*⁻ mutant and complementary strains by PCR: *oxyR-Like* sequence: amplification of the *oxyR-Like* gene fragment; upstream arm: amplification of the *oxyR-Like* upstream arm and the inserted Gm fragment; downstream arm: amplification of the *oxyR-Like* downstream arm and the inserted Gm fragment. (c) OxyR-Like expression analysis by western blotting in various cell strains. An anti-6His-OxyR-Like antibody was used as the primary antibody. Lanes *oxyR-Like*⁻ + *oxyR-Like*, Wild type and *oxyR-Like*⁻ correspond to the respective whole-cell supernatant fractions. 6His-OxyR-Like represents purified 6His-OxyR-Like protein.

All experiments were independently repeated three times to ensure their reproducibility.

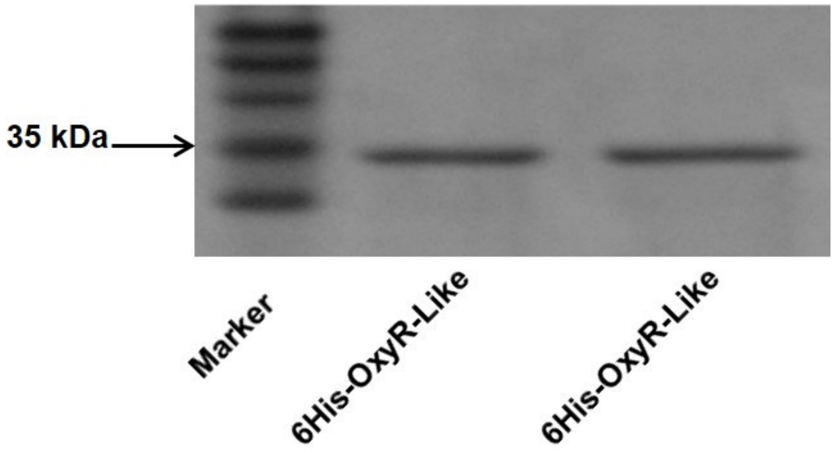

Supplementary Figure 2. Purification of the fusion protein 6His-OxyR-Like. 6His-OxyR-Like refers to purified OxyR-Like with a 6×His tag.

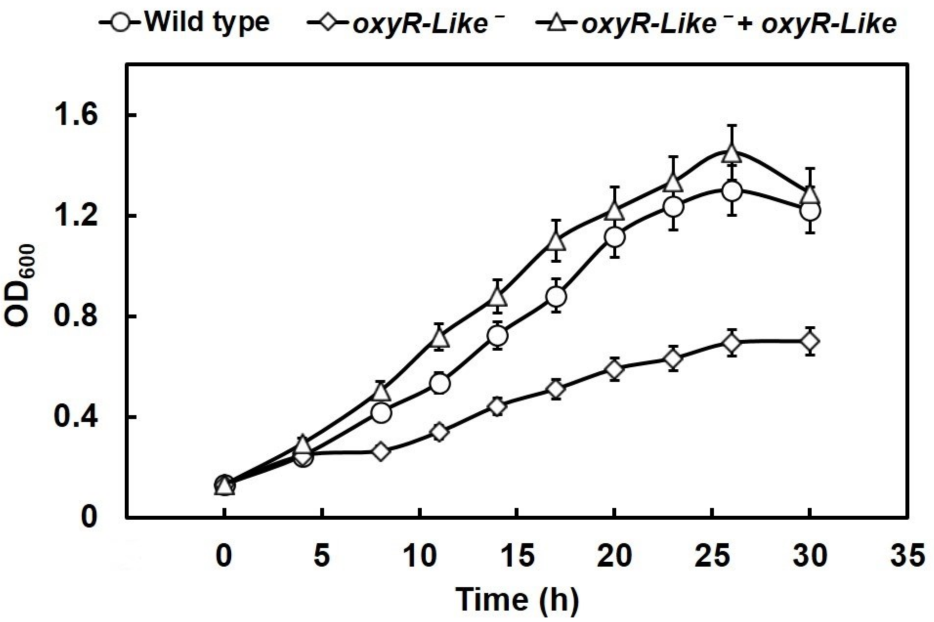

Supplementary Figure 3. Cell growth analysis of various strains. The cell density of the Wild type, *oxyR-Like*$^-$ and *oxyR-Like*$^-$ + *oxyR-Like* strains is represented by OD_{600}. All experiments were independently repeated three times to ensure their reproducibility.

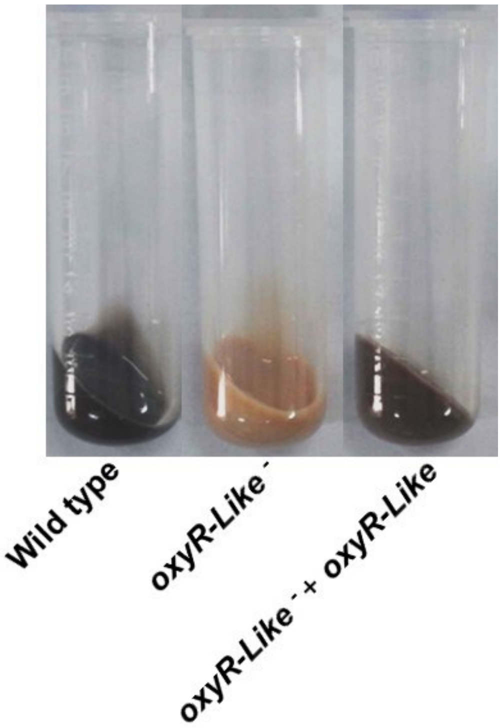

Supplementary Figure 4. Cell colors of various strains: Wild type, *oxyR-Like*$^{-}$ and *oxyR-Like*$^{-}$ + *oxyR-Like*.

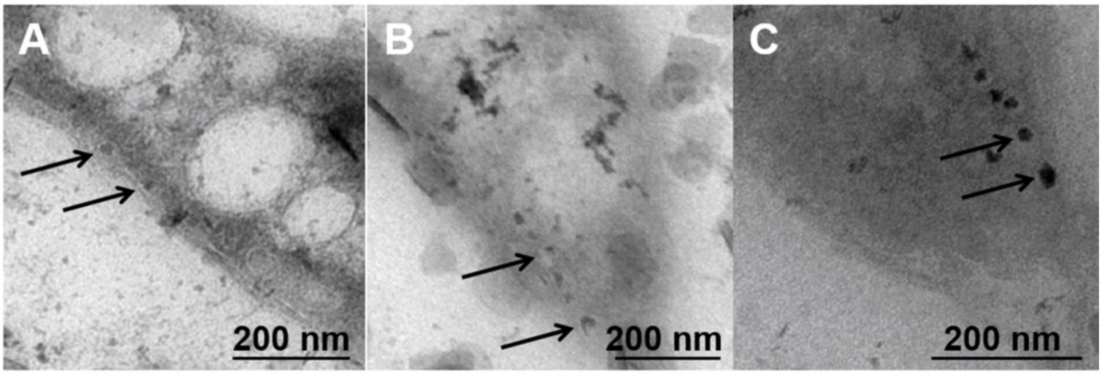

Supplementary Figure 5. TEM images of cryo-ultrathin sections of Wild type (A), *oxyR-Like*$^{-}$ (B) and *oxyR-Like*$^{-}$ + *oxyR-Like* (C), indicating that the magnetosome membranes occur in all strains (as arrows pointed).

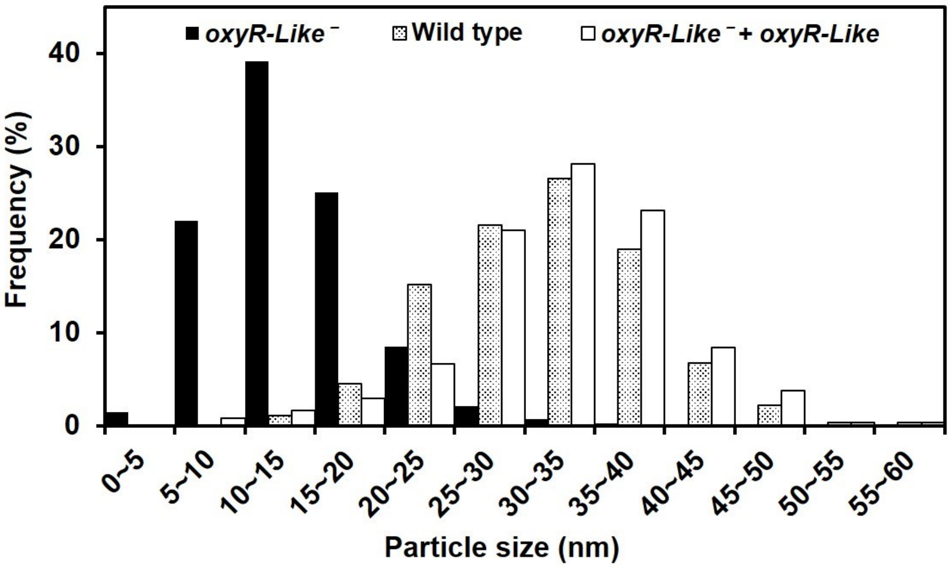

Supplementary Figure 6. The size distribution of magnetosomes in various strains: Wild type, *oxyR-Like*$^{-}$ and *oxyR-Like*$^{-}$ + *oxyR-Like*.

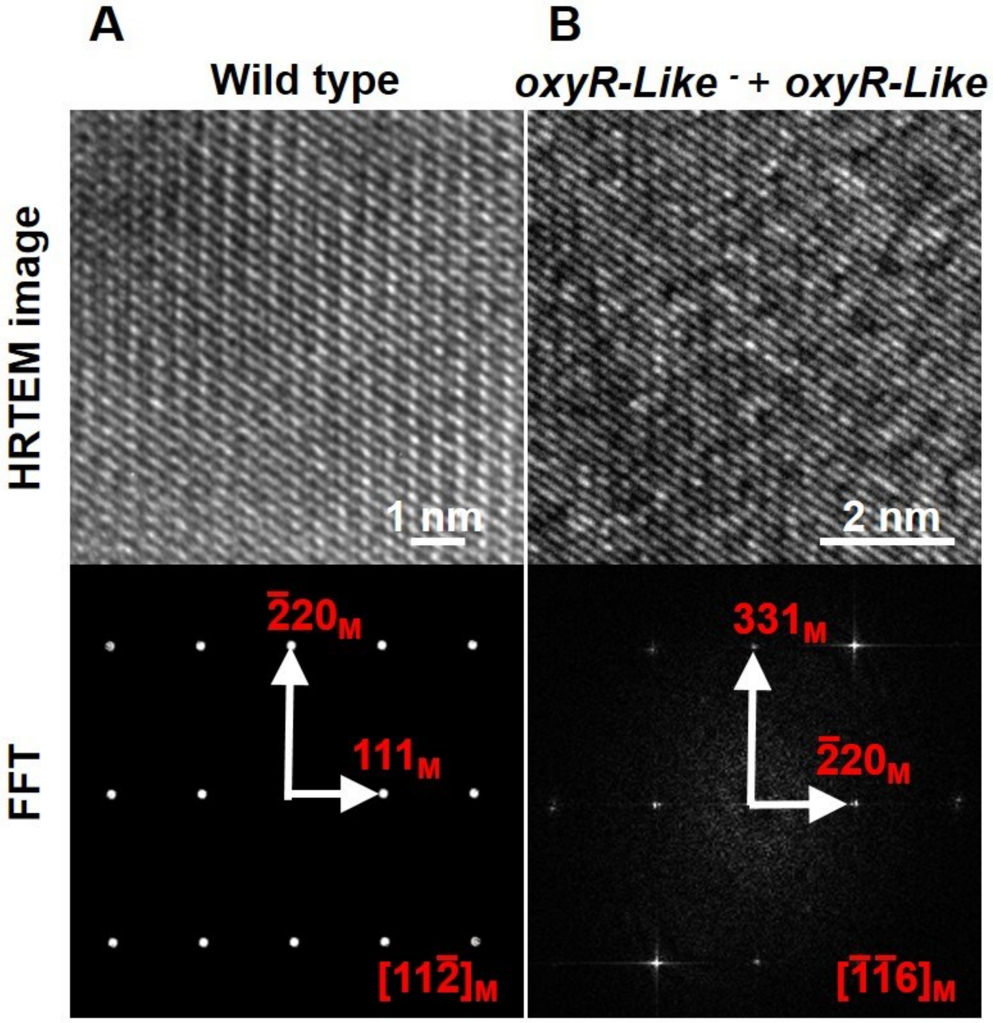

Supplementary Figure 7. High-resolution transmission electron microscopy (HRTEM) and fast Fourier transform (FFT) analyses of intracellular magnetosomes formed by Wild type (a) and oxyR-Like complementary strain *oxyR-Like*$^{-}$ + *oxyR-Like* (b).

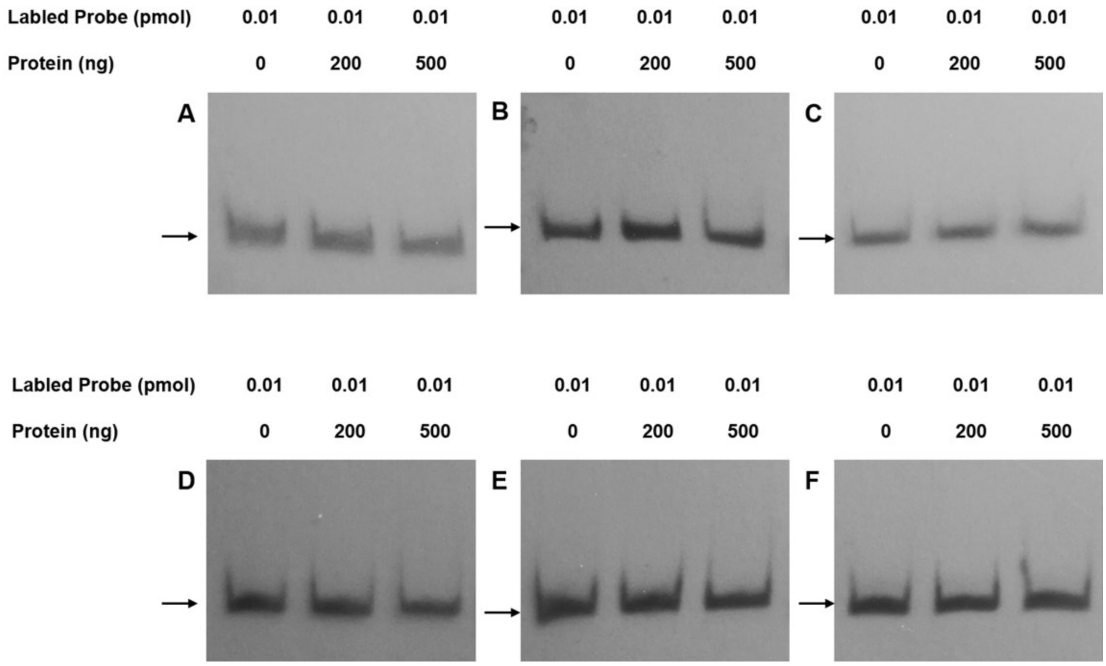

Supplementary Figure 8. OxyR-Like can not bind the promoter regions of ROS-eliminating genes. EMSA assay results indicate that OxyR-Like have not the ability to bind the promoter regions of *kanE* (MGMSRv-2_1681, A), *katG* (MGMSRv-2_1805, B), *bsaA* (MGMSRv-2_2769, C), *ahpC-0756* (MGMSRv-2_0756, D), *ahpC-2735* (MGMSRv-2_2735, E) *and sodB* (MGMSRv-2_1221, F). 200 ng and 500 ng of OxyR-Like protein were added to the binding system of each probe as suggested in the figure, respectively. The black arrows in the figure indicate the positions of free DNA probes.

Supplementary tables

Table S1. Bacterial strains and plasmids used in this study

Strains or plasimds	Relevant characteristics	source
**Strains**		
E. coli DH5α	*endA1 hsdR17* [r-m+] *supE44 thi-1 recA1 gyrA* [NalR] *relA relA1* Δ[*lacZYA-argF*] *U169 deoR* [Ø80Δ (*LacZ*) M15]	Novagen
E. coli DH5α-T-oxyR-Like-s	containing *oxyR*-Like-up arm-pMD18T; Amp ^{r}	this study
E. coli DH5α-T-oxyR-Like-x	containing *oxyR*-Like-down arm-pMD18T; Amp ^{r}	this study
E. coli DH5α-9TT-oxyR-Like	containing pPR9TT-oxyR-Like	this study
E. coli S17-1	*Thi endA recA hsdR* with RP4-2-Tc ::Mu-Km::Tn7 integrated in chromosome; Sm ^{r}, Tra $^{+}$	Novagen
E. coli S17-1-202-oxyR-Like	S17-1 containing pSUP202-*oxyR-Like* S-Gm-*oxyR-Like* X; Cm ^{r}, Amp ^{r}, Gm ^{r}	this study
E. coli S17-1-9TT-oxyR-Like	S17-1 containing pPR9TT-oxyR-Like; Nx ^{r}, Amp ^{r}, Cm ^{r}	this study
E. coli BL21(DE3)	F^{-}*ompT hdS$_{B}$* (rB^{-} mB^{-}) *gal dcm* (DE3), general purpose expression host	Novagen
E. coli BL21-OxyR-Like	BL21 containing pET28a-oxyR-Like; Km ^{r}	this study
WT-PR9TT	Wild-type containing pPR9TT; Nx ^{r}, Amp ^{r}, Cm ^{r}	this study
OxyR-Like-9TT	*oxyR-Like*-Deficient mutant containing pPR9TT; Nx ^{r}, Gm ^{r}, Amp ^{r}, Cm ^{r}	this study
OxyR-Like-C	The *oxyR-Like* complemented strains of *ΔoxyR-Like* mutant	this study
**Plasmids**		
pMD18-T	Linearized vector with T-overhang for direct cloning of PCR fragments with A-overhang, *lacZa*, ori, f1-origin, Amp ^{r}	TaKaRa
pMD18-oxyR	pMD18 carrying fragment of *oxyR-Like* gene; Amp ^{r}	this study
pMD18-oxyRs	pMD18 carrying fragment of *oxyR-Like*-uparmgene; Amp ^{r}	this study
pMD18-oxyRx	pMD18 carrying fragment of *oxyR-Like*-downarmgene; Amp ^{r}	this study
pET-28a(+)	Expression vector for *E. coli*, N-terminal 6×His-tag, Km ^{r}	Novagen
pET-28a-oxyR	expression the His-tag-N-terminal protein of OxyR-Like; Km ^{r}	this study
pUCGm	pUC19 derivative containing aacC1 (encoding Gm^{r} gene) Amp ^{r}, Gm ^{r}	Novagen
pSUP202	suicide vector for MSR-1; Cm ^{r}, Amp ^{r}, Tc ^{r}	Novagen
pSUOxyR-Like	pSUP202 derivative, carrying *oxyR-Like* flanking sequences and Gm ^{r} gene; Cm ^{r}, Amp ^{r}, Gm ^{r}	this study
pPR9TT	Broad-host range *lacZ* promoter probe vector; Amp ^{r}, Cm ^{r}	this study
pPROxyR-Like	pPR9TT carrying fragment of *oxyR-Like* and kanamycin promoter gene; Amp ^{r}, Cm ^{r}	this study
pUC4K	A vector carrying Amp ^{r}, Km ^{r}. Source of kanamycin promoter (PKm)	this study

Table S2. Primers used in this study

Name	Sequence	Description
oxyr-Like-uf	CACTGCGAGCTCTCAATCACGGACAACAGCCT (*Sac* I)	The 5′ flank sequence of *oxyR-Like*
oxyr-Like-ur	CATCGCGGATCCGGCGGAGTGAAACGACAGAA (*BamH* I)	The 5′ flank sequence of *oxyR-Like*
oxyr-Like-df	CATCCCAAGCTTCCATCAAGAAAGTCACTGGCA(*Hind* III)	The 3′flank sequence of *oxyR-Like*
oxy-Liker-dr	CACTGCGAGCTCAATACCGCCTGTTCGCTCA(*Sac* I)	The 3′flank sequence of *oxyR-Like*
coxyR-Like-f	CATCCCAAGCTTTACGTGGATGAGTTCGGCAA(*Hind*III)	Complementation of the Δ*oxyR-Like*
coxyR-Like-r	TCCAGGATCCCCATTAGGCTGTTGTCCGTGA(*BamH*I)	Complementation of the Δ*oxyR-Like*
kanp-f	TTCGAGCTCACCGAACTGAGATACCTACA (*Sac*I)	Kan promoter
kanp-r	GGATCCATAACACCCCTTGTATTACTGTT (*Bgl* II)	Kan promoter
OxyR-Like -P-f	GGAATTCCATATGATGATCAGCTTTCGCCAATTACAATGCC(*EcoR*I)	Expression of OxyR-Like protein
OxyR-Like -P-r	CCGCTCGAGTTACCGCCTGTGCACGCAAGCC(*Xho*I)	Expression of OxyR-Like protein
	**Primers used for EMSA**	
EMSA-oxyR-LikeF	GGGCGAAGAATGGACATC	Probe amplification of *oxyR-Like* promoter
EMSA-oxyR-LikeR	AGGCATTGTAATTGGCGAAA	Probe amplification of *oxyR-Like* promoter
EMSA-pdhF	TTTGCTCTGAATGGGTGC	Probe amplification of *pdh* promoter
EMSA-pdhR	CAGGGCAGGATGATGTGA	Probe amplification of *pdh* promoter
EMSA-kaeEF	TCTTGTATCAGGACGGCTC	Probe amplification of *katE* promoter
EMSA-katER	TGGGCTGTTGCTTGTCTT	Probe amplification of *katE* promoter
EMSA-katGF	GCGGCTACACCGAATACG	Probe amplification of *katG* promoter
EMSA-katGR	CGGCACTTGTTCCATGAA	Probe amplification of *katG* promoter
EMSA-bsaAF	CTTGATCCCGATAATCTGCTG	Probe amplification of *bsaA* promoter
EMSA-bsaAR	AGTCCAAGACAGCACCACT	Probe amplification of *bsaA* promoter
EMSA-ahpC-0756F	GAGTTGGAGCGGCTGTTG	Probe amplification of *ahpC-0756* promoter
EMSA-ahpC-0756R	TGAAGGGCTTGATTTCGG	Probe amplification of *ahpC-0756* promoter
EMSA-ahpC-2735F	CTTCATCCGCCAAGCACC	Probe amplification of *ahpC-2735* promoter
EMSA-ahpC-2735R	GGATTGACGGCATCGGAC	Probe amplification of *ahpC-2735* promoter
EMSA-sodBF	GGAATCCGCCATTTCTTC	Probe amplification of *sodB* promoter
EMSA-sodBR	GCGGTCTTGTCATAGGGA	Probe amplification of *sodB* promoter
	**Primers used for Foot printing**	
Foot-oxyR-LikeF	TCTCGTCACCTTCGTCCTT	Foot printing primers for *oxyR-Like* promoter
Foot-oxyR-LikeR	CCAGATGCTCTTCCAGTTG	Foot printing primers for *oxyR-Like* promoter
Foot-pdhF	TGGGCGGGTTCTGTGTTC	Foot printing primers for *pdh* promoter
Foot-pdhR	ATCTCCGCCGACTTGGCT	Foot printing primers for *pdh* promoter
	**Primers used for qRT-PCR**	
qPCR-pdhAF	CCAGGGTCAGGTTTACGAAAG	qRT-PCR to detect the expression of *pdhA*
qPCR-pdhAR	CGCTTGAACAATTCGATACCG	qRT-PCR to detect the expression of *pdhA*
qPCR-pdhBF	AGGTGATGGAAAAGGCTGG	qRT-PCR to detect the expression of *pdhB*
qPCR-pdhBR	GCGGTTGTACTTGGCATATTC	qRT-PCR to detect the expression of *pdhB*

qPCR-pdhCF	CCTATAAGCTGTCGGTCAACG	qRT-PCR to detect the expression of *pdhC*
qPCR-pdhCR	ACACGTCCACATCGGTATAAC	qRT-PCR to detect the expression of *pdhC*
qPCR-lpDF	GAAAATTCCAAGATTCCCGGC	qRT-PCR to detect the expression of *lpD*
qPCR-lpDR	CTTGACCTGATAGCCCTTCTC	qRT-PCR to detect the expression of *lpD*
qPCR-gltAF	GTTCTACCACGATTCCACCG	qRT-PCR to detect the expression of *gltA*
qPCR-gltAR	TTCTGCGGATACATGAACGG	qRT-PCR to detect the expression of *gltA*
qPCR-fumAF	CCGTCTGCTTGAAGTATTCCG	qRT-PCR to detect the expression of *fumA*
qPCR-fumAR	TGAAATCCGCAAGACCCTG	qRT-PCR to detect the expression of *fumA*
qPCR-fumCF	TCGTGATGTGCCAGCTTG	qRT-PCR to detect the expression of *fumC*
qPCR-fumCR	TTTCGAGACCCATTGCGG	qRT-PCR to detect the expression of *fumC*
qPCR-pckGF	GAAATGGCTGATGGTGATGTTG	qRT-PCR to detect the expression of *pckG*
qPCR-pckGR	GTATGGGTCAAAAGGTGCTG	qRT-PCR to detect the expression of *pckG*
qPCR-sdhCF	CCGTGGCTGAGATGGAAG	qRT-PCR to detect the expression of *sdhC*
qPCR-sdhCR	GATCGGTATGTTCGCCCTG	qRT-PCR to detect the expression of *sdhC*
qPCR-acnBF	GGTGATGTTGGAGGTCATGTAC	qRT-PCR to detect the expression of *acnB*
qPCR-acnBR	GGTCGTATCCTGGAAATCGAG	qRT-PCR to detect the expression of *acnB*
qPCR-sucAF	ACAGACGAACGCCATGAC	qRT-PCR to detect the expression of *sucA*
qPCR-sucAR	GACTTCAACATCAACCGCAAG	qRT-PCR to detect the expression of *sucA*
qPCR-sucCF	GCAGACCCAGATACAGTTCG	qRT-PCR to detect the expression of *sucC*
qPCR-sucCR	AGGGCAAGGAAGTCAAGC	qRT-PCR to detect the expression of *sucC*
qPCR-mdhF	GACCGACACCATGACCTTG	qRT-PCR to detect the expression of *mdh*
qPCR-mdhR	TCAATGCCCGTTACTCCG	qRT-PCR to detect the expression of *mdh*
qPCR-icdF	ATGCCGTACTTGTCGAACTC	qRT-PCR to detect the expression of *icd*
qPCR-icdR	ACACCATTCTCAAGGCCTATG	qRT-PCR to detect the expression of *icd*

Table S3 Crystallographic information of the crystals determined in this study.

Image identifier	Fig.4i-1	Fig.4ii-1	Fig.4iii-1	Fig.4iv-1	Fig. S7A	Fig. S7B
Projection axis	[22-1]	[01-2]	[11-1]	[01-1]	[11-2]	[-1-16]
Reflections	012 and 102	121 and 200	202 and 022	122 and 022	-220 and 111	331 and -220
Ratio measured between two sets of reciprocal spacings (referred to as r1 and r2)	1.03 r1 = 3.86 Å and r2 = 3.75 Å	1.25 r1 = 3.16 Å and r2 = 2.53 Å	1.02 r1 = 2.08 Å and r2 = 2.03 Å	0.85 r1 = 2.67 Å and r2 = 3.14 Å	0.6 r1 = 3.0 Å and r2 = 5.0 Å	0.67 r1 = 2.0 Å and r2 = 3.0 Å
Ratio between spacings for alpha-Fe_2O_3 (referred to as r1 and r2)	1.0 r1 = 3.68 Å and r2 = 3.68 Å		1 r1 = 2.08 Å and r2 = 2.08 Å			
Ratio between spacings for epsilon-Fe_2O_3 (referred to as r1 and r2)		1.23 r1 = 3.12 Å and r2 = 2.54 Å		0.84 r1 = 2.7 Å and r2 = 3.2 Å		
Ratio between spacings for magnetite (referred to as r1 and r2)					0.61 r1 = 2.97 Å and r2 = 4.84 Å	0.65 r1 = 1.93 Å and r2 = 2.97 Å
Angle measured between two sets of reciprocal spacings (degrees)	48	52	59.5	89	90	90
Angle between reciprocal lattice directions for alpha-Fe_2O_3	50		57			
Angle between reciprocal lattice directions for epsilon-Fe_2O_3		52		90		
Angle between reciprocal lattice directions for magnetite					90	90

For further crystallographic information, people can refer to pdf card as 33-0664 for alpha-Fe_2O_3, 65-3107 for magnetite and 52-1449 for epsilon-Fe_2O_3, respectively.
